# Using Psychophysiological Sensors to Assess Mental Workload During Web Browsing

**DOI:** 10.3390/s18020458

**Published:** 2018-02-03

**Authors:** Angel Jimenez-Molina, Cristian Retamal, Hernan Lira

**Affiliations:** 1 Department of Industrial Engineering, Faculty of Physical and Mathematical Sciences, University of Chile, Santiago 8370456, Chile; hlira@ing.uchile.cl; 2 Department of Electrical Engineering, Faculty of Physical and Mathematical Sciences, University of Chile, Santiago 8370448, Chile; cristian.retamal@ug.uchile.cl

**Keywords:** psychophysiological sensors, mental workload, Web browsing tasks, machine learning

## Abstract

Knowledge of the mental workload induced by a Web page is essential for improving users’ browsing experience. However, continuously assessing the mental workload during a browsing task is challenging. To address this issue, this paper leverages the correlation between stimuli and physiological responses, which are measured with high-frequency, non-invasive psychophysiological sensors during very short span windows. An experiment was conducted to identify levels of mental workload through the analysis of pupil dilation measured by an eye-tracking sensor. In addition, a method was developed to classify mental workload by appropriately combining different signals (electrodermal activity (EDA), electrocardiogram, photoplethysmo-graphy (PPG), electroencephalogram (EEG), temperature and pupil dilation) obtained with non-invasive psychophysiological sensors. The results show that the Web browsing task involves four levels of mental workload. Also, by combining all the sensors, the efficiency of the classification reaches 93.7%.

## 1. Introduction

Although Web applications are often justified in terms of increasing the productivity of human tasks, they sometimes have the opposite effect, interrupting, reducing the performance of, or increasing the mental workload of the user [1,2,3,4]. A typical task in which this phenomenon may occur is Web browsing. In this task, the user fixes her/his gaze on and between Web elements, i.e., graphic or textual areas of a Web page, such as news, commercial advertisements, and menus [5,6,7]. In cognitive psychology and ergonomics, mental workload refers to the amount of perceived effort induced by a particular task [8].

An important factor in measuring the effectiveness of a Web page is the user’s browsing experience. It has been shown that the higher the level of user’s browsing experience is, the lower the mental workload [3,4,9]. Continuously assessing the mental workload involved in browsing tasks entails measuring it either when the user fixes her attention on a Web element or when her gaze switches from one element to another. This assessment of mental workload can enhance the user’s browsing experience in many ways: for instance, automatically identifying the most suitable moments to proactively deliver content to the user or preventing irritating intrusions from the environment; keeping the Web page support interventions on stand-by and adapting graphic user interfaces in real time; and evaluating the likelihood of user’s abandonment, frustration or techno stress, among other benefits. In addition, instantaneous classification of mental workload would make it possible to detect short time windows of reduced cognitive burden to activate the delivery of different types of recommendations in a timely, unobtrusive manner, such as contextual news in newspaper portals or commercial advertisement pop-ups on various Web sites. In addition, it may be possible to enhance search tasks, for instance, for restaurants, flight tickets, or retail products, by providing relevant feedback to the search engine based on the user’s cognitive status [6].

To realize the above requirements, it is essential to address the challenge of automatically assessing the mental workload in a continuous fashion while the user is engaged in browsing, that is, in real time, with high frequency and using very short time windows.

Many studies have focused on classifying mental workload in general by capturing and processing data using ever less invasive psychophysiological sensors [10,11,12,13,14]. This method is founded on the empirical demonstration of the correlation existing between psychological stimuli and physiological responses triggered by the nervous system. Moreover, mental workload has been shown to vary frequently within a short time span [15,16]. 

Although considerable research has been devoted to assessing mental workload on the scale of hours and minutes by using data extracted from psychophysiological sensors, less attention has been paid to time windows lasting seconds or less, such as when a user fixes her gaze on a Web element. Indeed, Bailey et al. [17] have recently proved that moments of reduced mental workload occur while the user’s attention is transiting from one task to another. However, this was shown only for coarse-grained tasks, such as selecting a travel route among alternatives presented in a graphic interface or classifying a list of emails into various categories [17].

In this paper, the capabilities of psychophysiological sensors are leveraged to research the possibility of assessing mental workload in real time during a browsing task. This paper thus attempts to answer the following research questions:

- RQ1: Is it possible to identify levels with regard to a user’s mental workload within very short time windows (order of milliseconds) based on psychophysiological signals recorded during a Web browsing task?
- RQ2: Is it possible to accurately classify in real time a user’s mental workload, both when her gaze is fixed on a Web element and when her gaze is transiting from one Web element to another, by combining different non-invasive psychophysiological sensors?

In addition, based on the findings of Bailey et al. [17], this paper attempts to prove the following hypothesis:

- *H1: Mental workload is significantly* *smaller when the user’s attention is switching from one Web element to another than when she is focused on a Web element.*

To answer these research questions and prove the stated hypothesis, an experiment was conducted in which 61 users performed a normal Web browsing task in front of a computer screen while their psychophysiological responses were measured by different sensors and recorded in a database. The gold standard with regard to answering RQ1 is pupil diameter because several previous studies have shown that, under controlled illumination conditions, this psychophysiological response is a valid and reliable indicator of mental workload [17,18,19,20,21,22,23]. Using clustering methods, this paper shows that, by processing the pupil dilation response, four levels of mental workload can be identified.

However, measuring pupil dilation with an eye tracker is not a realistic and practical method to classify mental workload, for example, in the open air, because it requires constant and controlled illumination conditions. Thus, in this paper, more practical and less invasive sensors are assessed to measure other psychophysiological responses, such as heart rate (HR), electrodermal activity (EDA), body temperature, and electrocardiogram (ECG). The electroencephalogram (EEG) sensor is also assessed because there have been important advances in the construction of portable EEGs and in algorithms to reduce motion-related artifacts [24,25]. It is expected that before long, there will be EEG devices that only capture brain waves from the areas of the brain relevant to the assessment of mental workload, making them less invasive [26].

This paper shows that, using all the sensors and efficiently processing their signals using a multi-layer perceptron, among other models, mental workload can be classified as proposed in RQ2. Furthermore, the hypothesis that mental workload is significantly smaller when the user’s attention is switching from one Web element to another than when she is focused on a Web element is confirmed.

The contributions of this paper include: (i) identifying the different levels of mental workload required for Web browsing through the processing and analysis of pupil dilation measured by an eye-tracking sensor; (ii) developing a method for appropriately combining non-invasive psychophysiological sensors to classify real-time mental workload in small time windows with high accuracy based on the behavior of the user’s gaze in a Web browsing task; and (iii) leaving open the possibility of using gaze shifts from one Web element to another as the most appropriate time to provide the user with recommendations, for example.

This paper is organized as follows: Section 2 provides the background required to understand this research. Section 3 presents the related literature. The experiment conducted is described in Section 4, as well as the data processing and the machine learning methods applied to the data. The results are presented in Section 5 and are discussed in Section 6, while Section 7 concludes the paper.

## 2. Background

### 2.1. Assessment Methods

Cognitive resources are assets used by cognition to think, remember, make decisions, solve problems, or coordinate movements, such as perception, attention, short- and long-term memory, and motor control [27,28]. According to Navon et al. [29], these resources underlying human learning and information processing are limited [30].

Wickens [8], in his multiple resource theory, suggests that these resources can be used in parallel for multiple tasks, using several resources at once. However, when task demand is high, the resources allocated to that task are not available for another task if the same mental resources are required at the same stage of processing. Excessive use, moreover, can cause a state of overload known as cognitive resource depletion [31]. This overload means that the brain is unable to process new information, resulting in processing and/or execution errors [32].

Mental workload results from the different levels of resource demand, depending on the parallel tasks that the person is performing [8,15,16,33]. Excessive resource demand can cause distraction, increase errors, generate stress and frustration, and reduce the ability to undertake mental planning, problem solving, or decision-making [34,35]. One example is the distraction caused by unwelcome advertisements on a Web page while the user is browsing. In this case, the intermingling of the browsing task with the intrusion of commercial advertisements forces the user to divide attention and allocate cognitive resources to the new stimulus.

Traditionally, mental workload has been assessed in different situations using subjective methods [10] based on surveys, auto-perception scales, or think-aloud protocols [36,37,38]. These methods are applied after the user has already finished the task, and the assessment of the mental workload depends of the user’s final perception [39]. Therefore, these methods are constrained by the reporting bias introduced by relying on past memories and by the problem of ecological validity based on observing responses to hypothetical scenarios rather than behaviors in a real setting [40]. In addition, the static nature of these methods makes them unfit for real-time evaluation. The most widespread example of this method is the NASA Task Load Index, which measures the mental and physical performance, as well as the effort and frustration, of the user [41]. 

Performance-based methods have also been used, which measure indicators generated during task execution, such as the percentage of correct responses or execution time [3,10,11]. In this method, the user needs to be engaged in only one task. Its major restriction is the difficulty of assessing mental workload in near real time.

The attempts to find objective indicators to measure mental workload in real time are based on collecting contextual information, which can be captured mainly using psychophysiological sensors [42,43,44]. Indeed, there is ample empirical evidence in psychophysiology showing that some physiological responses are directly related to psychological factors such as stress, mental workload, and emotions [45,46,47]. That is, there is a correlation between the physiological responses triggered by the nervous system and psychological stimuli.

Psychophysiological responses are controlled by the autonomic nervous system (ANS), which regulates and coordinates bodily processes such as digestion, temperature, blood pressure, and many aspects of emotional behavior [48]. These actions occur independently of the conscious control of the individual. The ANS includes the sympathetic nervous system (SNS) and parasympathetic nervous system (PNS). The SNS controls actions required in emergency situations, such as stress and movement. It can cause heart rate acceleration, pupil dilation, and increased blood flow to the muscles, sweating, and muscle tension. The PNS controls the functions related to rest, repair, and relaxation of the body. The responses elicited by this system include a decrease in heart rate and blood pressure, stimulation of the digestive system, and pupillary contraction, among others [45,46].

### 2.2. Psychophysiological Measurements

There are different types of methods to measure psychophysiological responses elicited complementarily by the SNS and PNS [49]. For instance, the device for tracking gaze is the eye tracker. It consists of a camera typically positioned below the computer screen that works according to the “corneal-reflection/pupil-center” method, which consists of recording the centre of the pupil to identify the gaze position and recording the reflection of infrared lights [50]. It also allows the measurement of the variation of the pupil diameter. Pupillography measures changes in pupil size, which can be attributed to both parasympathetic inhibition, which explains the first dilation phase, and sympathetic activation, which explains the subsequent contraction phase [51,52]. Although pupil dilation can be triggered by a light reflex caused by changes in environment illumination or by a proximity or accommodation reflex to improve visual focus, it can also be caused by a psychosensory reflex associated with the cognitive or emotional engagement of the person while exposed to any sensory stimulus [53]. In contrast to changes in the two previous reflexes, changes in pupil size in this case are subtler, so a high-precision device or eye tracker is required for their detection [54].

Nevertheless, some shortcomings with pupillometrics need to be taken into account. For instance, response delays can reach up to 1000 ms, which may invalidate the work with short time windows; pupil variations can be due to multiple factors, such as exhaustion, stimulants and gaze shifts, among others. The eye tracker is also used for tracking the eye to determine gaze position or movements within a scene, including two relevant measurements:

- *Fixations*: moments during which the gaze is relatively fixed or focused. They occur because sharp vision is only possible within a small area in the human eye called the fovea. It is useful to determine when eye fixation occurs because, in most cases, it coincides with attention.
- *Saccades*: rapid eye movements or jumps from one fixation point to another. Saccades follow a pattern (or trajectory) depending on several factors: what is currently being looked at, visual target tracking, experience, and emotions.

**Figure 1:**
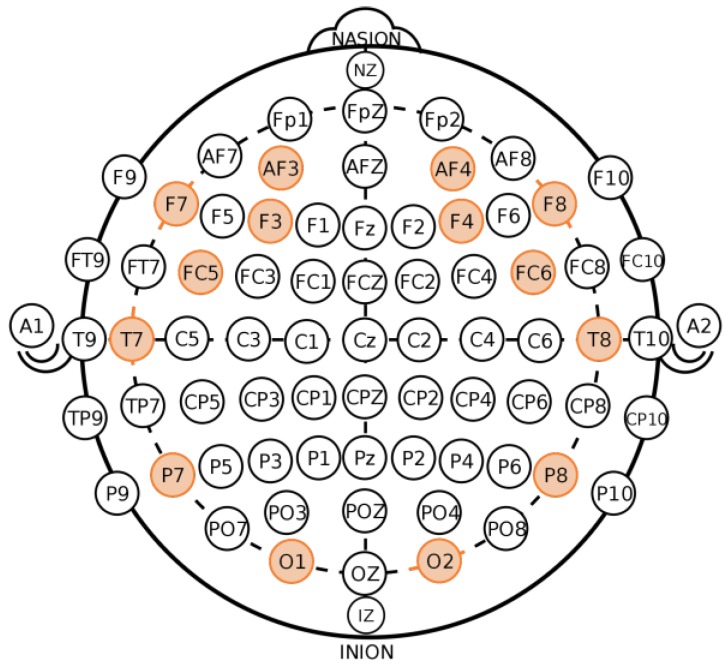
The 10–20 system of electroencephalogram electrodes. Highlighted the 14 electrodes used in this paper.

Another set of psychophysiological measurements is obtained by electroencephalography. This is based on recording the electrical activity of the brain measured on the scalp. The device used is the EEG, which measures the voltage resulting from changes in ionic current flow within the neurons of the brain, produced by the brain’s synaptic activity. The EEG signal is a blend of different subjacent frequencies, which represents different cognitive or affective states. For its capture, it is used, among others, the 10–20 distribution of electrodes located on the skull (see Figure 1). Each electrode is named with a letter and a number. The first refers to a specific region of the brain—frontal lobe (F), temporal lobe (T), center (C), occipital lobe (O)— while the second indicates its position. If this number is even, it represents the right side, if odd, the left side. 

There are five major brain waves: delta (1–4 Hz), theta (4–8 Hz), alpha (8–12 Hz), beta (12–25 Hz), and gamma (approximately 25 Hz). The alpha band is suppressed during mental or bodily activities with open eyes. The suppression of the alpha band is a sign of mental activity and commitment to the task. This means that the brain is coordinating attention resources and focusing on the task. The alpha band is generated in the occipital, parietal and posterior temporal areas of the brain.

The theta band correlates with the difficulty of mental operations, for example during periods of focused attention or information gathering, processing and learning and during memory recall. It has been found that the frequency of the theta band becomes more prominent when the difficulty of the task increases. This band can be obtained from the whole cortex, which indicates that it is generated by a wide network that involves the prefrontal, central, parietal and temporal cortices.

There is evidence that the most relevant bands when it comes to distinguishing cognitive load are the alpha and theta bands in the parietal and frontal lobes, respectively, suppressing the first and increasing the second [10,55,56,57].

In general, these bands are used limited to the EEG channels that correspond to frontal and parietal lobes (F3, F4, F7, F8, P7 and P8). However, in [57] it is indicated that, although the oscillations of the alpha and theta bands reflect changes in cognitive load and memory performance, it is important to define the alpha and theta band for each subject starting of the peak frequency of its alpha band, named as the Individual Alpha Frequency. That is, the cutting frequencies are not the same for each person. Despite this, there is literature that uses the standard EEG bands to classify cognitive load with good results [58,59,60,61]. 

EDA is a psychophysiological response that can be assessed by measuring changes in the electrical properties of the skin. Skin conductivity varies with changes in skin moisture (sweat) and may reveal changes in the SNS. EDA is also known as galvanic skin response (GSR), and it is inexpensive to assess, easily captured, and robust. It is measured by attaching one or two electrodes usually to the fingers or toes. It is an indicator of psychological and physiological arousal. When arousal increases, there is an increase in sweat gland activity, decreasing electrical resistance, and thus increasing conductivity. In addition, it serves to identify emotional states. 

EDA has two components named tonic and phasic. The tonic component or base signal varies slowly, presenting slight changes in the scale of 10–100 s and sets basic skin conductance. The rise and decay of the signal changes constantly within the same subject, depending on its hydration, dry skin or autonomic regulation. This component can differ highly between subjects. The phasic component or conductive response of the skin is above the tonic component and shows significantly faster alterations. The signal is sensitive to specific emotional stimulus events, which induce peaks that occur between 1–5 s after the start of the stimulus.

The cardiovascular system is particularly interesting for psychophysiology because it is highly sensitive to neurological processes and psychological factors such as stress. It is regulated by the ANS, which produces patterns of electrical activity that are fundamental for psychophysiological measurements [45]. Several studies associate changes in cardiac activity with psychological phenomena, such as mental work, perception, attention, problem solving, and signal detection [63].

An ECG is used to measure the electrical activity of the heart, using at least three electrodes attached to the chest. The electrodes collect the necessary data with regard to the electric waves that describe the cardiac cycle, based on which the HR or its variation (HRV) are obtained.

The human body constantly exchanges heat with the environment as part of the process of self-regulation to maintain homeostasis (internal balance of the body). Body temperature increases and decreases in relation to the energy exchanged. The regulation of blood flow to the skin and thermal radiation is considered a function of the ANS [64]. Studies conducted in this field, according to Genno et al. (1997) [65], suggest that skin temperature has potential as a psychophysiological measure of the individual.

## 3. Literature Review

This paper focuses on the measurement of mental workload while the user browses a Web site in front of his or her personal computer. The literature in this regard is scant. Thus, to start studying the measurement of mental workloads in various domains and to help understand the methodology associated with this type of research, this section focuses on two main points: the assessment of mental workload using psychophysiological sensors in general and the measurement of mental workload in Web environments.

### 3.1. Assessment of Mental Workload with Psychophysiological Sensors

A relevant study for this paper is that by Bailey et al. [17] who develop psychophysiological measures to assess the effect of interruptions on the performance of a person executing a task. They establish that interruption involves considerable negative effects, such as increased time to complete the task [35], a wider range of errors [66], additional efforts in decision-making [67] and mood changes such as increased frustration and anxiety [68,69,70]. For example, when an interruption occurs at a random time while performing a major task, the time to completion can increase by up to 30%, up to twice as many errors can be committed, and user displeasure doubles, in contrast to when the interruption occurs at a pre-programmed time. Therefore, Bailey et al. empirically find that interruptions may have a lower cost if they occur at a time of low mental workload, hypothesizing that this may occur at the boundaries between subtasks when executing the general task [71]. As a test method, they assess mental workload by pupil dilation in three different tasks that include respective subtasks. The first task consists of assessing two different routes between two cities on a monitor; the user must measure the distance and cost of the routes, tabulate the data, and, finally, discriminate and choose the shortest and most economical route. In the second task, the user must edit a document and correct spelling at three levels of complexity (editing a word, editing two words, and editing a complete sentence). The third task entails classifying nine emails involving explicit issues (low complexity) and ambiguous issues (high complexity) into four categories. Each of these scenarios is applied to 24 people (seven women) between 19 and 50 years of age. The main conclusions of the study are as follows: (i) mental workload varies during the execution of the three tasks, (ii) the mental workload decreases when performing subtasks compared to the general task, and (iii) different subtasks demand different levels of mental workload based on their complexity.

Other studies focus on training classifiers to process psychophysiological signal data in a time window in order to predict whether the load associated with a specific task is high or low [72]. For example, Haapalainen et al. [11] measure the mental workloads of basic tasks such as the resolution of problems on a monitor, visual perception, and cognitive speed by using an eye-tracking device, EEG, ECG, heat flow, and rate measurements. As a result, they find that ECG and heat flow together distinguish between tasks of high and low cognitive demand with 80% precision.

Fritz et al. [10] seek to verify whether psychophysiological sensors are useful in measuring the difficulty of a computer code comprehension task with various levels of difficulty. The tasks are performed by software developers, who are monitored using an eye tracker and an electroencephalogram. Fritz et al. use the Beta/(Alpha+Theta) ratio based on the evidence that beta increases with task execution, theta is deleted, and alpha is blocked. The models obtained classify task difficulty with 85% accuracy.

Shi et al. [73] assess stress and arousal levels by measuring EDA for increasing levels of difficulty. The experiment consists of a transition interface in which the participants must respond to the requirements in three scenarios: (1) using gestures and speaking, (2) only speaking, and (3) only using gestures. The difficulty varies depending on level of visual complexity, number of entities, number of distractors, time limit, and number of actions to complete. The results indicate that there is a significant increase in the EDA signal as task difficulty increases.

Nourbakhsh et al. [74] confirm the effectiveness of EDA in discriminating between the difficulty of eight arithmetic tasks with four levels of difficulty. In addition, as an extension of the previous study, Nourbakhsh et al. measure mental workload using EDA changes and the number of blinks obtained from an eye-tracking device. The experiment is the same as in the previous study. This time, by combining both sensors, 75% precision is achieved for the lowest level of difficulty.

Xu et al. [75] show that mental workload can be measured by pupil dilation if illumination changes. The experiment consists of arithmetic tasks that vary in difficulty depending on the number of digits.

In Ikehara et al. [12], an eye-tracking device, a pressure sensor for the mouse, an EDA sensor, and a pulse oximeter (for measuring HR and level of oxygen in the blood) are used. The experiment consists of selecting on a screen the fractions whose value is less than 1/3. There are two levels of difficulty in the experiment. The results indicate that EDA and pupil dilation have the greatest statistical significance in terms of detecting task difficulty.

Using an elastic neural network, Hogervost et al. [13] find that the best performance is obtained when EEG is combined with pupil dilation (91% accuracy) and when EEG is combined with peripheral physiology (89%); with EEG alone, they obtain 86% accuracy. In addition, using only the measurement of the electrode located in the Pz position (central parietal area of the head), they obtain 88% accuracy.

### 3.2. Assessment of Mental Workload in Web Environments

Although the study of users’ cognitive responses during Web browsing is an intriguing area, it remains little explored. Indeed, one of the few studies on the topic is that by Albers [3], who examines how mental workload theory applies to the design of Web sites using the tapping test method, which measures mental workload by focusing on performance. As in all the examples using this approach, the tapping test adds an additional secondary task to the main one, measuring the performance of the participant to determine the level of mental workload induced. In this case, the main task is to browse two Web sites sequentially—with implicit mental workload controlled by design—and answer questions aloud in relation to the Web pages, while the secondary task is to rhythmically keep tapping per second. As mental workload increases, tapping begins to fall slowly and lose the rhythm, even losing it completely when there is cognitive overload. However, implementing a secondary task as required by this method prevents from generating a realistic scenario for the user and does not allow real-time measurement.

The most recent research regarding the observation of Web users’ experience involves the measurement of their behavior as a reaction to different stimuli, such as notifications, and allows us to predict the user’s response according to Navalpakkam and Churchill [76]. By comparing mouse pointer movement to eye tracking, they are able to determine a more user-friendly layout for a Web site, which improves the effectiveness of the notification. Finally, they conclude that gaze and mouse movement patterns contain important information in terms of assessing the user’s status, determining if they are distracted from the assigned task or striving to fulfill it. The correlation between eye movements and mouse pointer movement predicts a Web user’s different psycho-emotional states. They also conclude that the user is more likely to pay attention to notifications when they vary in position on the Web site rather than when they are fixed.

Table 1 summarizes the discussion of the literature by assessing to what extent each related work encompasses the major concerns of this paper: (1) capacity to assess mental workload during very short time windows; (2) capacity to provide mental workload classification results near to real-time measurement; (3) evaluation based on Web browsing tasks and (4) use of multiple psychophysiological sensors. 

As conclusion, the measurement of mental workload using psychophysiological signals has been tested for a varied set of tasks. In addition, studies have investigated how mental workload is related to the design of a Web page. However, the above mentioned research provides no evidence regarding assessment of mental workload while browsing a Web site using multiple psychophysiological measures. There is also no reference to time overhead to determine how feasible it is to implement near real-time measurement.

## 4. Materials and Methods

### 4.1. Participants

The initial experimental group includes 61 participants (19 women and 42 men), aged between 19 and 35 years (mean age = 23.8 years, SD = 3.2 years), all engineering students at the University of Chile, recruited through the institutional news Web application. None of them suffered from cardiovascular diseases or was taking medications that could have affected their normal behavior. All of them were familiar with browsing tasks. Each session had a duration of approximately 60 min. The final experimental group is composed of 53 people. Eight participants were rejected due to various problems during signal measurement and processing.

This research has the approval of the Research Ethics Committee at the Faculty of Physical and Mathematical Sciences at the University of Chile. In addition, all of the participants read an informed consent and agreed on signing it. The consent contained information about the procedure, purpose of the experiments, voluntary participation, right to decline to participate at any moment, how to access the research results and researchers’ information.

### 4.2. Psychophysiological Sensors

Psychophysiological sensors have the advantage that measurements do not depend on the user’s perception and are not under the control of the user. 

In addition, they are becoming less intrusive and allow tasks to be performed in various scenarios, giving greater ecological validity to the experiments. They also allow real-time data capture [10,45]. 

**Figure 2:**
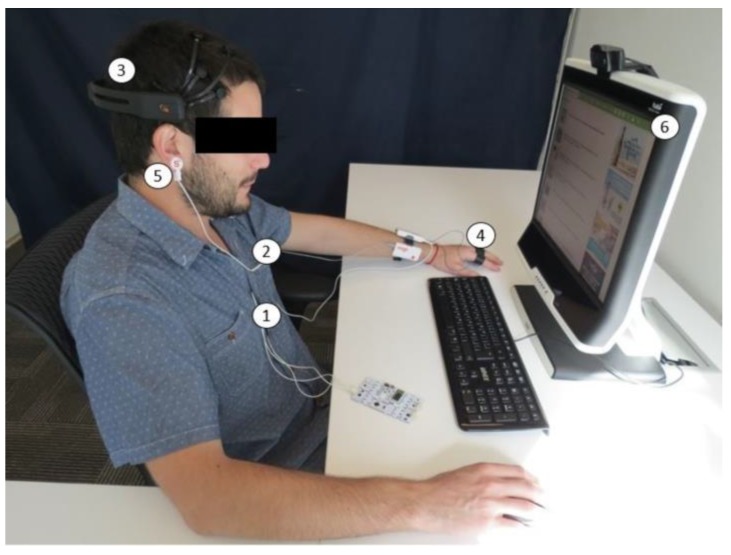
Participant with the sensors runs the experiment. The sensors are: (**1**) ECG, (**2**) axillary temperature, (**3**) EEG, (**4**) EDA, (**5**) PPG and (**6**) eye tracker.

For data acquisition, the following sensors were used: *GSR*+, optical pulse sensor, and Bridge Amplifier + unit, all from the Shimmer [77]; ECG BITalino [78]; EEG Emotiv Epoc [79]; and Tobii T120 Eye Tracker [80]. Figure 2 shows an example of a volunteer outfitted with all the sensors.

To measure the EDA and HR signals, the Shimmer GSR+ unit sensor was used with a sampling frequency of 120 Hz. The position of the electrodes for measuring the EDA was the palm area of the proximal phalanx of the index and ring fingers of the left hand [81]. The optical sensor that functions as a photoplethysmograph (PPG) was attached to the lobe of the right ear [82]. The Shimmer Bridge Amplifier + unit sensor with a sampling frequency of 50 Hz was used to measure body temperature. The sensor was applied under the right armpit. This sensor was synchronized with the EDA and pulse sensors using a base provided by Shimmer together with the Consensys software.

The BITalino BioMedical Development All-in-One Board with a sampling frequency of 1000 Hz was used to measure the ECG. The configuration of the three electrodes followed the lead II standard [83,84]. Before applying the electrodes, the skin was prepared by wiping it with alcohol to remove grease and impurities to reduce noise. In addition, an ECG gel was used. OpenSignals evolution software provided by the manufacturer was used [85].

To measure the EEG, the Emotiv EPOC EEG sensor with a sampling frequency of 128 Hz was used. The sensor was attached to the head, positioning the reference sensors first. To improve the conduction of the electrical signals of the brain, each electrode was previously hydrated. To capture the data and verify that the sensor was properly applied, the Emotiv Xavier Testbench software provided by the manufacturer was used.

The Tobii T120 Eye Tracker with a sampling frequency of 120 Hz was used to measure pupil dilation and for eye tracking. Tobii Studio software was used for calibration and to perform data collection [86]

### 4.3. Task Design

**Figure 3:**
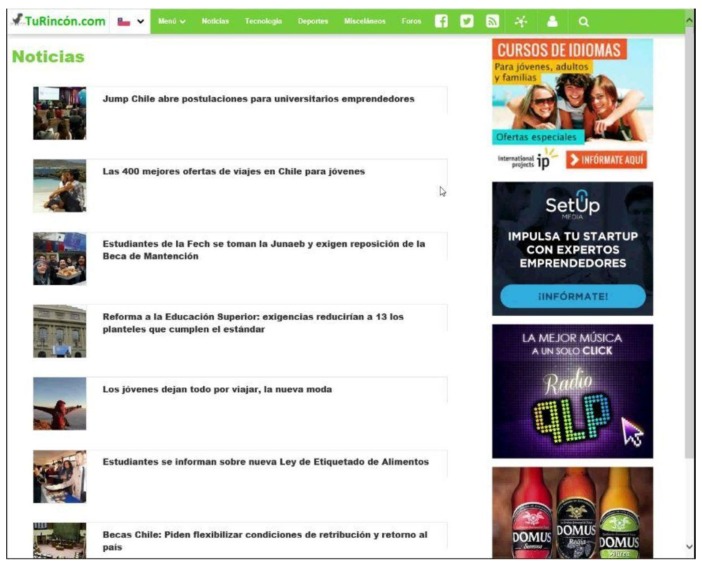
Example of a dummy Web page used for the experiment.

A fictitious Web site was created whose basic configuration is shown in Figure 3. This layout of the Web elements was maintained through all the experiment. The elements within the Web site were seven news headings with their respective representative image, four rectangular advertisements, a typical navigation bar with a menu, the logo of the page in the upper left corner, and a bar at the bottom of the page.

In order to minimize possible factors affecting pupil dilation other than changes in mental workload, we ensured that the luminosity level of the room keep constant. A physically isolated experimental room was used to maintain the experimental configuration and the environment constant for all participants. In addition, the room did not receive any sunlight, to avoid the effects of infrared light on measurements and to maintain constant illumination conditions that do not affect pupil diameter measurements [62]. In addition, we verified that the brightness of the Web page’s interface does not suffer significant changes during the experimental session that may affect the dilation of the pupil.

**Figure 4:**
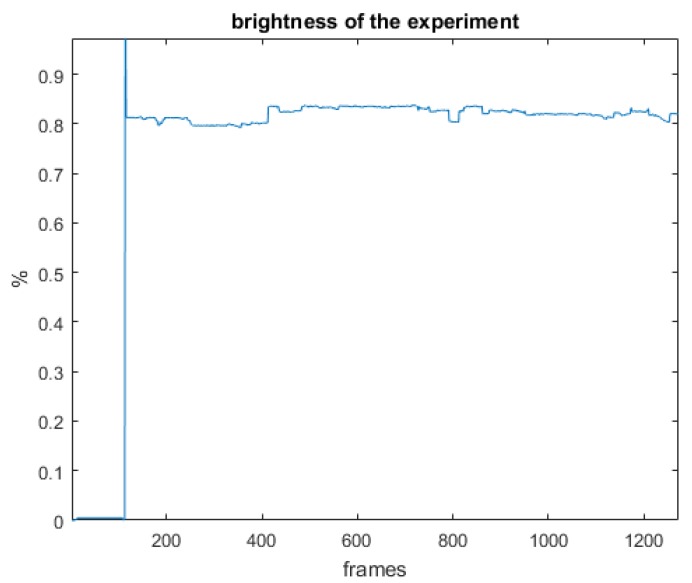
Brightness of the Web page for each frame during a experimental session.

In fact, an approximation to the Web page brightness was obtained as follows. A pre-experimental session was conducted from which the video of the Web page was obtained for the duration of the session. The image of each frame of the video was transformed to grayscale, calculating the average value of the brightness of all the pixels. In effect, the values of the grayscale pixels range from 0 (black color) to 255 (white color). Taking the measure of brightness as a percentage value, the average of the values of the pixels was calculated and divided by 255, thus obtaining the percentage of brightness in the image of each frame. Figure 4 shows the percentage of brightness in each frame. The initial fall corresponds to a black color interface before the deployment of the experiment instructions. The initial peak, in turn, corresponds to the delivery of the instructions with a white background. It is worth noting that when navigation on the Web page begins, the brightness in each frame undergoes negligible variations, remaining practically constant during the experimental session.

Another factor knowns to cause pupil dilation differences is the effect on arousal that the own Web page’s content or other stimuli may produce. According to Ward and Marsden [87] it can be stated that a Web browsing experiment does not have contentious elements that cause significant variations in arousal if the rate of variation of the EDA is maintained at percentages of 3–4.5%. Therefore, like the analysis of the brightness of the Web page, the EDA signal of each participant obtained in their respective experimental session was analyzed in time windows of 500 ms. For the analysis performed, an average of −0.8102% EDA variation rate with a standard deviation of 1.4944 was obtained for the 52 valid participants. In this way, it is validated that, like the luminosity, the arousal factor does not reach values that may significantly alter pupillary dilation.

### 4.4. Experimental Procedure

Each participant was tested individually at the laboratory. As soon as each participant arrived in the experimental room, the experiment was explained to him/her, and he/she was asked to read and sign the informed consent, as well as a questionnaire to get their basic anonymous information. The participant was seated in front of the screen, and the sensors were connected in the following order: ECG, axillary temperature, EEG, EDA, and PPG; then the eye tracker was calibrated with the help of the participant (Figure 2). 

Prior to the tests, each user underwent a relaxation period consisting of the visualization of three four-minute videos of landscapes with background instrumental music. Then, the participant was asked to take deep breaths for one minute with eyes closed and with soft background instrumental music. This procedure aimed to eliminate the Hawthorne effect—modification in the behavior of the subjects due to their awareness of being studied—and physiological effects similar to the “white coat” effect in measured signals [88]. Next, the participant was asked to maintain a fixed posture, sitting in front of the computer, without moving the head or the left hand, where the sensors were connected. The instructions were that the user could freely browse the Web site for as long as they wanted, indicate when they wanted to finish, and that after the browsing he/she would have to fill out a questionnaire regarding the Web page content. Finally, all sensors were removed from the participant, and he/she was asked to not tell others about the experimental procedure.

### 4.5. Data Analysis

#### 4.5.1. Time Window Definition

In this paper, mental workload is assessed during two time windows:

- *Active window*: Time during which the user fixes her gaze on a specific area of interest (AoI), which may correspond to a news headline, an advertisement, or the menu bar of the Web site.
- *Transition window*: Time that elapses while the user is not fixing her gaze on any of the areas of interest. It can be a transition between two elements or towards the same element.

**Figure 5:**
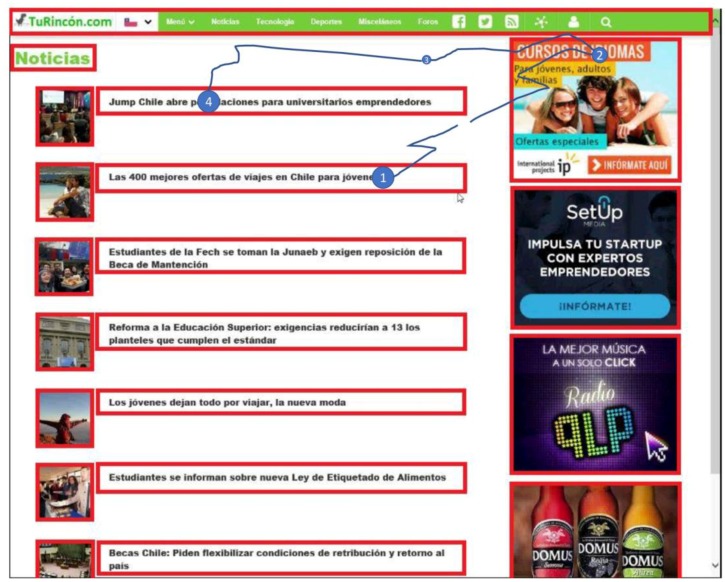
Example of active window and transition window.

As illustrated in Figure 5, the red rectangles represent the studied AoIs; the blue circles represent fixations, which size varies in accordance with the fixation time and the blue lines represent the saccades. Thus, the time a fixation is into an AoI pertains to an active window. The time between two fixations, such as fixation one and fixation two, pertains to a transition window. Note that the transition window between fixation two and four add the fixation three, which does not fall into any AoI. 

To discriminate between types of windows, the data file exported from the Tobii Studio program generates a column showing the AoI that the participant is inspecting for each sample. It discriminates between three values: when the user is not looking at the screen—inactive—, when the user is looking at a certain AoI—active window, and when the user’s gaze is directed outside the AoI—which is considered a transition window.

A long minimum time of 500 ms is set to define a valid time window. This is based on the research of Loyola et al. [89], who assesses the identification of key Web elements in a Web site using eye tracking. This time span is selected to avoid possible contamination of the pupil signal by the analysis of a previous object. Time windows below the threshold are not considered for analysis and are therefore deleted. When the same Web element is analyzed before and after a deleted window, the two segments are joined, generating a window of greater length.

#### 4.5.2. Data Preprocessing

The data exported from Tobii Studio contains the diameter of the left pupil, the diameter of the right pupil (both in millimeters), and the validation of the reliability of the capture of each pupil between 0—high reliability—and four—the eye was not detected. On average for all participants and considering only valid windows, the reliability of the capture of the left pupil is 0.2469, and that of the right pupil is 0.22036; these are reliable values to validate the capture of pupil diameter data. As these values are an average for all the participants, the pupil data with the highest level of reliability are selected for each sample [10].

Next, signal distortion artifacts, such as saccades and blinks, are eliminated. A column in the extracted data shows if the sample is a fixation or a saccade, and this information is used to filter saccades. Furthermore, a linear interpolation between the values of the blinks detected is used. In addition, a Blackman window with a cut-off frequency of 2 Hz is applied as a low-pass filter.

Normalization of the pupil signal is performed using the baseline value obtained from the average calculation of the pupil area 500 ms before subjecting the participant to the stimulus. To calculate the area it is assumed that the pupil is a circumference. Then, the base area is subtracted from the area of the signal during the experiment to calculate the dilation or constriction of the pupil [90]. The z-score is applied to the resulting signal in order to normalize the data and allow comparison between subjects.

In the same way, the baseline is calculated for all other signals considering the 500 ms prior to the stimulus and subtracting the baseline average from the experimental signal. Z-score is also applied in order to compare signals between subjects. Then, each signal is processed as explained below.

EDA raw data provides the values of electric resistance of the skin in kilohms [kΩ]. To reduce noise and eliminate motion artifacts, two procedures are performed: first, a strict instruction is given to each participant not to move the hand or fingers where the electrodes are attached, and second, the signal is filtered with a low-pass cut-off frequency of 5 Hz. Furthermore, on the recommendation of the literature [91], capture resolution is reduced without risk of data loss. The EDA signal measured with a sampling frequency of 120 Hz is reduced to 10 samples per second. The phasic component is extracted by applying a median filter with a window width of ±4 and subtracting the average of the current sample [91]. This component allows the detection of peaks of the EDA signal. With slow transitions, the phasic component does not show major variations.

Regarding the electrocardiogram, the raw data yield values that must be transformed to millivolts [mV]. The processing of this signal consists of using a low-pass filter with a cut-off frequency of 100 Hz and applying the fast Fourier transform to obtain the characteristic shape.

The raw data of the PPG yield signal values in millivolts [mV]. From this signal, it is possible to obtain the HR. Previously, the PPG signal is processed using a low-pass filter with a cut-off frequency of 16 Hz with a Blackman window, obtaining a cleaner signal. Then, HR is obtained via the following steps: first, the peaks must be found; second, the time between them is substracted (∆t in [miliseconds/pulse]); third, they are converted from hundredths to seconds and from [seconds/pulse] to [pulses/second], which is then multiplied by 60 to convert to [*beats*/*minute*]. This is resume in the Equation (1):

(1) $$\mathrm{HR}=\frac{60}{∆t\cdot 100}\;\left[\frac{beats}{minute}\right]$$

The raw data yield body temperature values in degrees Celsius. The processing of this signal consists of using a low-pass filter with a cut-off frequency of 1 Hz, as concluded based on the data collection in Haapalainen et al. [11].

The EEG signal is subject to a wide variety of artifacts and noise [92,93]. Among the elements that cause artifacts are blinking, oculomotor activity, head movements, facial expressions that add noise due to the muscle electrical signal, and movement of the electrodes, among others. To eliminate the effect of head swinging, a high-pass filter with a cut-off frequency of 0.5 Hz is used. In addition, a low-pass filter with a cut-off frequency of 40 Hz is used to eliminate noise from the electrical grid (50–60 Hz). To eliminate outliers and decrease the effect of the blinking artifact a Hampel filter is used [94].

#### 4.5.3. Feature Extraction

Feature extraction is performed based on time windows. Since signals have different scales, to be comparable objects, it is necessary to standardize them before extracting characteristics from them, as proposed by Guyon et al. [95]. To perform standardization, the classical (x−μ)/σ form is used, where x is the vector corresponding to the signal, and μ and σ are the mean and the standard deviation of the signal, respectively. 

A total of 44 characteristics pertaining to the different signals are extracted: two from pupil dilation, six from EDA, two from body temperature, three from ECG, three from PPG-HR, and two from each of the 14 EEG channels. Table 2 shows a summary of the characteristics, following which the obtained characteristics are presented in more detail.

Because it has been proven that pupillary response is an important indicator of the mental effort required to solve a task, it is selected as the gold standard by which to cluster windows and generate labels for cognitive levels. There are clustering cases in the literature regarding the development of Web tasks such as the study of Loyola et al. [89]. The selected characteristics are the mean and variance of the pupil diameter of the eye that displays greater reliability in its measurement.

Based on the findings of Nourbakhsh [74] and Shi et al. [73], the following characteristics are extracted from the processed EDA signal: accumulated normalized data, mean as a function of normalized time, and spectral power without normalized continuous component. Equation (2) shows the calculation of the normalized EDA signal. Each point in time t is added, where i corresponds to the participant, k and m is the total number of tasks; m=3 in this case:

(2) $${\mathrm{EDA}}_{normalized}\left(i,k,t\right)=\frac{\mathrm{EDA}\left(i,k,t\right)}{\frac{1}{m}\sum_{j=1}^{m}\sum_{t=1}^{T_{ij}}\mathrm{EDA}\left(i,j,t\right)}$$

Therefore, the data for each participant are normalized by dividing the task signal by the mean value of all the tasks for the subject. Then, the accumulated *EDA* characteristics are calculated as shown in Equation (3) and mean *EDA* is calculated according to Equation (4), where T is the total time for all the tasks:

(3) $${\mathrm{EDA}}_{accumulated}\left(i,k\right)=\sum_{t}{\mathrm{EDA}}_{normalized}\left(i,k,t\right)$$

(4) $${\mathrm{EDA}}_{average}\left(i,k\right)=\frac{\sum_{t}{\mathrm{EDA}}_{normalized}\left(i,k,t\right)}{T}$$

The following characteristics are extracted from the phasic component obtained: number of peaks, maximum modulus, and average of the phasic component of the window [10].

Based on the proposal by Haapalainen et al. [11], the following characteristics are selected for the *ECG* signal: median, mean, and variance of the *ECG* median absolute deviation (*ECG_MAD*), calculated using Equation (5):

(5) $$ECG\_MAD=\left|{\mathrm{ECG}}_{i}-median\left(\mathrm{ECG}\right)\right|$$

The characteristics of the heart rate obtained from the PPG signal are selected based on the time domain characteristics used in Betella [96]. These are the mean, standard deviation, and root mean square of HR. Based on the proposal by Haapalainen et al. [11], the median and mean of the temperature are selected.

For the EEG signal, there are two main approaches: event-related potential (ERP) analysis and time-frequency signal analysis. The latter is selected because it is more closely related to the psychophysiological and structural processes of the brain [92]. It is used to study emotional-cognitive states in particular and is more advisable when studying a limited period or a relatively low amount of data, as is the case of the time-window study of this paper [97]. Among the different ways of analyzing the EEG signal in time-frequency are frequency bands with Fourier transform, Morlet wavelets, and Hilbert transform. All three show similar results according to Cohen [98]. Thus, the option of the Hilbert Transform (ℋ{eeg}(t)) is selected, which has the advantage of greater control over frequency filtering. The Equation (6) shows this transform:

(6) $$\hat{eeg}=ℋ\left\{eeg\right\}\left(t\right)=\left(h*eeg\right)\left(t\right)=\frac{1}{\pi }\overset{\infty }{\underset{-\infty }{\int }}\frac{eeg\left(\tau \right)}{t-\tau }d\tau$$

where h(t)=1/πt, eeg is the EEG signal and $\hat{eeg}$ is the resulting analytic signal. Before applying this transform, a bandpass filter between 2 and 15 Hz is used to center the study in the theta (4–8 Hz) and alpha (8–12 Hz) frequency bands. These are related to states of mental activity and relaxation, respectively, where theta increases and alpha is suppressed when there is mental workload [97]. A complex signal called the “analytical signal” is then obtained, from which two characteristics are extracted. This is performed for each of the 14 channels of the EEG signal.

#### 4.5.4. Clustering

Clustering is performed for all participants to determine how many levels of mental workload the users present based on the measurement of pupil diameter. The database is labeled after ascertaining these levels. The pupil diameter data is used after the baseline correction and, as mentioned previously, the z-score standardization is performed with the aim of including all the participants in the analysis, establishing the cluster boundaries for all of them. In Loyola et al. [89], the k-means method is used. 

**Figure 6:**
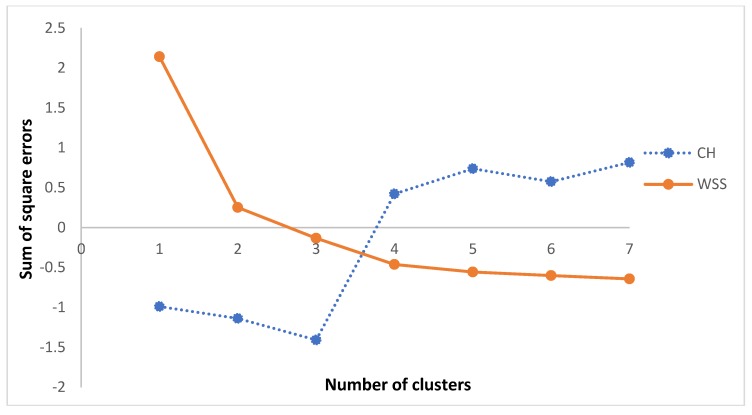
Optimal number of clusters according to the intersection method of CH and WSS curves for all participants.

**Figure 7:**
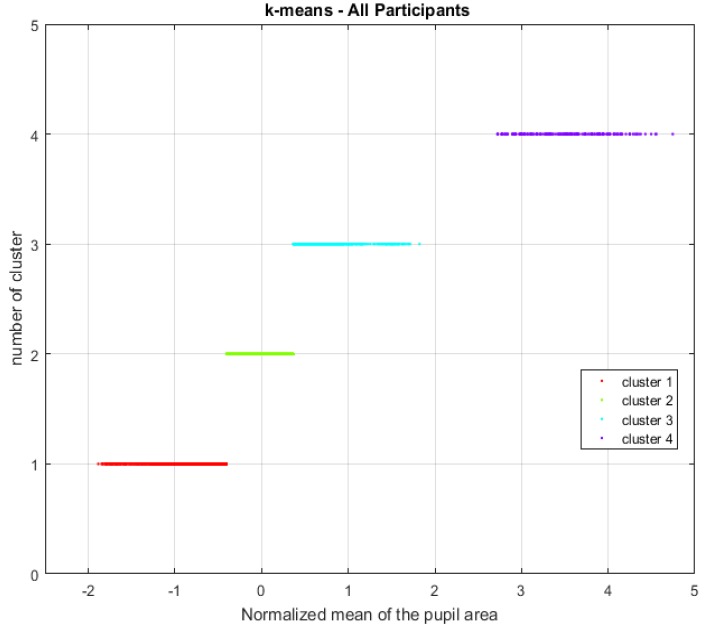
Optimal grouping of time windows according to their level of cognitive load considering all participants.

Because an overestimation or underestimation of the number K of clusters affects the quality of the cluster, the optimal value of clusters is sought. The K value is tested from two onwards to obtain two curves. The index of Calinski and Harabasz (CH) and the internal measure of cohesion of the sum of the squares within the group (WSS) are selected to this end [99,100,101]. The stop rule is the value closest to the point where the curves intersect. Figure 6 shows the curves obtained through this methodology, where the optimal value is generated at K=4. Visually, the grouping can be validated considering Figure 7.

The Jaccard coefficient obtained using the bootstrap method is used as an external criterion for validating clusters, which assesses how stable the cluster is [99,100]. Values between 0.6 and 0.75 indicate that the group is measuring a pattern in the data, but there is no certainty as to which points should be grouped. Groups with stability values above approximately 0.85 can be considered highly stable. 

For this study, the bootstrap method confirms that K = 4 is the optimal value since Jaccard coefficients are the best compared to other cluster numbers. The Jaccard indices are 0.79 (cluster 1), 0.72 (cluster 2), 0.75 (cluster 3) and 0.88 (cluster 4). Considering all the valid participants, this result shows that there are four levels of mental workload validated with acceptable cohesion indices (RQ1). 

#### 4.5.5. Feature Selection and Applied Machine Learning Models

A Feature Selection is performed in order to improve the efficiency and time costs of the classification. There is evidence in the literature regarding the use of the random forest and recursive feature elimination (RF-RFE) method for the selection of characteristics with good results when applied to the classification of mental workload with EEG signals [102]. This combines recursive elimination with random forest, that is, a set of decision trees that assesses features and generates a ranking following a score criterion. This method is executed with all the features extracted from signals with the clustered data for all the participants. Table 3 shows the subset of features selected.

To perform the classification, a training set is first generated with 70% of the observations and then a test set with the remaining 30%. To avoid biases, a 10-fold cross validation is performed in which the classes are distributed uniformly within each set. In addition, they are randomly selected while maintaining the proportions. 

Three classification models are applied: Multinomial Logistic Regression (m-LR), Multi-class Support Vector Machine (m-SVM) and multi-layer perceptron (MLP). Since the applied normalization validates a between-subject analysis, each classification result is obtained from the average resulting from executing each classification model 100 times. 

As an initial approach using the dataset in a classification problem, we perform a m-LR and am-SVM, since there are four classes of cognitive workload to classify. Binary logistic regression and support vector machine are commonly used in classification problems and, in particular, both have been used in previous studies with psychophysiological sensors. For instance, Fritz et al. [10] apply and SVM algorithm to classify cognitive workload using EEG, EDA and pupil dilation. For an independent variable with more factors, m-LR and m-SVM are less common approaches in the field. In particular, for m-LR we use a Softmax function with four outcomes and for m-SVM we implement an algorithm that build a classifier and compares it against the rest iteratively and choose the class with the greatest margin. 

Finally, we implement a MLP that uses as input all the features extracted from the signals without the need to use the RF-RFE algorithm. The programmed MLP has two hidden layers with 1000 neurons each and 500 epochs, with a rectified linear activation function, as used by Hinton [103]. The key, according to Hinton, to avoid overfitting is to include a 50% dropout for each layer, which prevents artificial neurons from co-adapting to training data.

Thus, each neuron in the hidden layers is omitted at random from the network with a probability of 0.5. In addition, another method added to avoid model overfitting is the $L_{1}$ and $L_{2}$ regularization method as a linear combination, as shown in Equation (7). For this, the objective function for the artificial neural network is defined as L(W,B|j), where W represents the weight matrix and B the column of bias vectors for each training example.

(7) $$L^{\prime }\left(W,B|j\right)=L\left(W,B|j\right)+\lambda_{1}R_{1}\left(W,B|j\right)+\lambda_{2}R_{2}\left(W,B|j\right)$$

where the values of $\lambda_{1}$ and $\lambda_{2}$ are parameters that weight the relative contribution of the penalty terms $R_{1}$ and $R_{2}$ (rule $L_{1}$ and $L_{2}$, respectively) in relation to the objective function L(W,B|j). The values of $\lambda_{1}=10^{-5}$ and $\lambda_{2}=10^{-5}$ are determined as recommended in the H20 manual [104].

## 5. Results

### 5.1. Statistical Analysis

The hypothesis that there is a decrease in mental workload in the transition time windows between the analysis time windows of one Web element and another is proposed. To verify the hypothesis, the mean pupil diameter within each window is selected as our gold standard. The objective is to determine if the mean pupil diameter varies depending on whether it is in an active window or in a transition object. An analysis of variance with repeated measures (ANOVA-RM) is performed since all participants perform every task of the experiment. For the analysis, the complete universe of windows of all the participants is considered. 

As a result, a p-value=0.00963 is obtained with a 95% confidence interval, so the null hypothesis is rejected. In addition, as shown in Table 4, mean pupil diameter in the transition windows is smaller than in the active windows. Therefore, it is concluded for the data as a whole that the difference between mean pupil diameter in the active windows and the transition windows is statistically significant and that the diameter is smaller in the transition windows (H1).

#### Baseline Statistical Analysis

In order to verify if the baseline correction performed in the pupil diameter is accurate for each participant, it is necessary to evaluate if there exists a significant difference between the original pupil diameter data and the baseline corrected pupil diameter data. With that aim, we perform a Shapiro test for each participant to verify normality. For each one of the participants the null hypothesis is rejected, which implies that it is necessary to apply a test for a generic distribution. Therefore, we apply the non-parametric Wilcoxon test for each participant. The null hypothesis tested is that the means of the original data and the baseline corrected data are the same. As a result, for all participants (except participant 50, which is eliminated), the null hypothesis is rejected which implies that the means of both are distinct. Then, our manipulation is valid. 

### 5.2. Classification

In Table 5 we show the results using the three classifiers (m-LR, m-SVM and MLP) using all the features extracted from signals and m-SVM using the selected features from RF-RFE algorithm. The evaluation metrics that we present are accuracy, recall and precision. Also we calculate the Kappa statistic to compare the expected results with the results of each model. Kappa statistic compares the observed accuracy, number of instances correctly classified in the confusion matrix through the used classifier, with the expected accuracy, accuracy that any random classifier would get using the number of elements that belong to each class. This statistic is especially useful to conclude if the imbalance of classes produce a bias classification and therefore, metrics like the accuracy would be distorted. 

The worst results are given by the m-LR model with 51.42% of accuracy and a Kappa statistic value of 5.92%. This result indicates that the model only tries to classify one class, so the difference between the observed and expected accuracy is large.

Next, the m-SVM model has better evaluation metrics with 66.48% of accuracy using all the features extracted from signals and 70.03% using only the subset of features from the RF-RFE algorithm. In these cases, recall and precision are similar to the accuracy and also the Kappa statistic, which implies that the imbalance of classes has a minor impact. 

The results are notoriously improved using the MLP classifier, with an accuracy of 93.7% for all the participants, 95.28% and 92.06% of recall and precision respectively. Kappa statistic is also an excellent metric in this case, with a value of 91.24%. In this case, the imbalance of clusters has a minor impact as well.

### 5.3. Evaluating Psychophysiological Sensors

To assess the performance of each sensor, the MLP that obtains the best results with all the sensors is selected as a supervised learning model. Table 6 shows the results of assessing the performance of each sensor separately. The sensor with the best performance is EEG, with 70.91% accuracy in the classification. The other sensors separately have a very low level of classification accuracy.

Combinations of the three sensors with higher performance are tested: EEG, EDA, and PPG (HR). As shown in Table 6, the combinations with EEG provide the best results. The combination with the highest performance is EDA, PPG (HR), and EEG, with 86.27%.

An important difference between the EEG sensor and the others is that it allows the extraction of a greater number of characteristics because the 14 electrodes contribute two characteristics each, for a total of 28 characteristics. This factor may explain the superior performance of this sensor compared to the rest. Therefore, it is concluded that it is possible to obtain good classification results for this experimental design with less than five sensors, even only with the EEG (RQ2). The temperature sensor and the ECG can thus be discarded.

### 5.4. Evaluating Time Overhead

For mental workload classification in a Web browsing task to lead to a real application, processing must be sufficiently fast, given that the time windows considered have a minimum length of 500 ms. Table 7 shows the classification time for each model that yielded the best results. 

## 6. Discussion

The results of the statistical analysis determine that pupil diameter in the transition time windows is statistically and significantly lower than in the active windows. Given the correlation between pupil dilation and mental workload broadly accepted in the literature, it is determined that there is a decrease in mental workload in the time windows between the analysis of one Web element and another (H1).

A possible application of the H1 validation is the generation of recommendation systems that support the user during Web browsing according to her interest, that is, when she is not cognitively overloaded. This is applicable, for example, to retail applications, advertisement or even productivity applications.

Regarding the assessment of the psychophysiological sensors to estimate mental workload during a Web browsing task, with the exception of the EEG, the signals of the sensors used do not provide an appropriate level of classification by themselves. This finding is aligned with what has been found in the literature about mental workload assessment by using psychophysiological sensors, although in different domains. We are unaware of any studies that provide the answer to this issue. Nevertheless, the combinations of signals with the EEG signals stand out, obtaining high performance results.

It is necessary to remark the importance of the normalization of all participants’ signals through their respective baseline. This allows us to perform a statistical analysis and to apply clustering and classification models to all the participants in the study, minimizing the different measures within the subjects due to the variance of biological conditions. In the best scenario, classifiers perform with very high accuracy, precision, recall and kappa statistics in comparison with previous studies in the field. This result may open the opportunity of applying psychophysiological signals in several domains with accurate results. 

In addition to these results, the experimental design is a contribution to the field since the task was designed with the aim of taking one more step to bring it closer to real situations and thus, approach to future applications. Previous studies structure the entire flow of a task by determining the input and output of the same, which is a situation that lacks ecological validity and is not applicable to reality. Further, as those tasks are highly structured, in past studies it is possible to apply subjective tests to assess cognitive workload as an alternative measure of it. In this study, in which the task is designed more dynamically it is not possible to apply this type of separated assessments since subjects are biased with tasks occurred prior to the actual one, so the answers are not accurate. One of the limitations of this work is not having labeled Web elements in the Web page, where each label had been identified in advance. As future work, it is necessary to improve this method for mental workload evaluation during Web browsing tasks, with the aim of having a separated measure to ensure that mental workload is really being measured with the analysis of psychophysiological signals.

As shown in Section 4.3, one of the aims of the task design was to control external factors that could affect the cognitive workload assessment. The main emphasis is given in known factors such as brightness, changes in luminance and arousal. In particular, conditions like brightness and luminance changes are well controlled due to the experimental room specially conditioned for this purpose. Also, the task and the experimenter behavior are designed to focus on mental workload, avoiding another physical or psychological responses. It was shown that these external factors do not have impact in pupil dilation. Despite this fact, we think that in future studies, more work has to be done in this aspect in order to accurately evaluate the experimental design. 

## 7. Conclusions

The study of human behavior and physiology when performing human-computer interactions activities is complex due to the multiple factors that affect each person in their performance and behavior with regard to this class of tasks. This paper assesses the behavior of a user in the simple task of browsing through a fictitious Web page created specifically for this study, using psychophysiological sensors.

It is shown that for the complete data set, that is, considering the complete universe of windows of all the participants, pupil diameter—as a measure of mental workload—is significantly lower in the transition windows than in the active windows. Therefore, patterns of low mental workload states are identified, and the hypothesis (H1) that it is indeed possible to measure mental workload in Web browsing activities and, moreover, that the mental workload of the user decreases in the transition from the analysis of one Web element to another while browsing is verified.

The unsupervised model of k-means analysis is applied to the mean of pupil dilation, based on which the Web browsing task involves four levels of mental workload. Thus, it is concluded that there are several mental workload states that can be determined (RQ1).

To classify levels of mental workload, three different models are used—m-LR, m-SVM and MLP—(RQ2). Among the multiple obtained results, it is worth noting that if the EEG is combined with the PPG and EDA, the kappa statistic of the classification reaches 80.72%. By using all the sensors this performance raises to 91.24% (RQ2).

In terms of future lines of research, it is proposed to use the data to study Web users’ mood behavior together with their cognitive behavior. In addition, it is proposed to focus the research on the EEG sensor, which showed superior performance, using other analytical approaches, such as wavelets and/or ERP, to determine the most relevant involved brain areas. In addition, we aim to improve the experiment design by defining browsing tasks with different levels of difficulty.

## Figures and Tables

**Table 1:** Related work analysis.

Reference	Small Time Windows	Real Time	Web Browsing Tasks	Multiple Psychophysiological Sensors
[15]	Partially. Time window of 23.7 s.	Yes	No. Desktop-based tasks.	Yes. Eye tracker, EEG, ECG, heat flux and HR.
[14]	Partially. Time windows between 5 s and 60 s.	Yes	No. Coding tasks.	Yes. Eye tracker and EEG.
[34,37]	Partially. Time windows of 30 s.	Yes	No. Arithmetic tasks	Yes. EDA and blink.
[16]	No. Time windows between 60 s and 70 s.	Yes	No. Arithmetic tasks	Yes. Eye tracker, EDA, pulse-oximeter, mouse pressure sensor.
[17]	No. Time windows of 2 min.	Yes	No. N-back task.	Yes. EEG, EDA, respiration, ECG, eye tracker.
[18]	Yes. Time windows of 550 ms.	Yes	Partially. Choosing a route, correcting spelling and classifying emails tasks.	No. Only pupillary dilation.
[3]	Not applicable.	No	Yes	No. Measurement of mental workload by tapping test.
[36]	No. Time windows between 100 s and 120 s.	Yes	Yes	No. Only eye tracking.
[38]	Yes. Time windows between 300 ms and 600 ms.	Yes	Yes	No. Only pupillary dilation.

**Table 2:** Features extracted by each signal.

Signals	Extracted Features
Pupil	mean of area
EDA	Accumulated data, average as a function of time and spectral power
Phasic	Average, absolute value of the maximum, number of peaks
ECG	Mean, median, variance of ECGMAD (average absolute deviation)
PPG(HR)	Mean, standard deviation, RMS of HR
T	Mean, median
EEG	Power and phase of the analytical signal obtained with the Transf. of Hilbert

**Table 3:** Selected features with the RFE method for all participants.

Signal	Selected features
EDA	Accumulated data Spectral power
Temperature	Mean
PPG	Mean HR Root Mean Square (RMS) of HR
EEG	Power channel 5(T7) Power channel 9(P8) Power channel 11(FC6) Power channel 12(F4)

**Table 4:** Standardized means of pupillary diameter for transition and active windows.

Factor	Mean	Standard Deviation
Transition	−0.0201	0.951
Active	0.0629	1.115

**Table 5:** Results of classification using different models.

Model	Accuracy (%)	Recall (%)	Precision (%)	Kappa (%)
m-LR	51.42	48.71	46.86	5.92
m-SVM	66.48	63.21	66.71	57.49
m-SVM + RFE	70.03	65.99	68.79	65.14
MLP	93.7	95.28	92.06	91.24

**Table 6:** Summary of per sensor classification results for MLP with 1000 neurons in each hidden layer and 500 epochs.

Sensors	Accuracy (%)	Recall (%)	Precision (%)	Kappa (%)
All	93.7	95.28	92.06	91.24
EDA	35.7	41.5	26.62	2.31
T	35.66	21.27	25.02	0.04
ECG	34.75	26.48	25.39	0.617
PPG	34.71	20.70	25.13	0.3
EEG	70.91	82.03	65.09	58.36
EDA + PPG	37.11	54.48	28.39	5.23
EDA + EEG	80.95	87.34	77.23	73.07
PPG + EEG	77.72	85.49	72.9	68.36
EDA + PPG + EEG	86.27	90.4	83.65	80.72

**Table 7:** Testing time for models.

Model and Sensor Combination	Mean [sec]	Standard Deviation [sec]
m-LR	0.00073	0.0010
m-SVM	0.00668	0.0025
M-SVM + RFE	0.00124	0.0048
MLP	1.47667	0.6091
MLP EEG	1.12334	0.0057
MLP EDA + PPG	1.10667	0.0057
MLP EDA + EEG	1.14667	0.0115
MLP PPG + EEG	1.13667	0.0057
MLP EDA + PPG + EEG	1.11667	0.0115

## Supplementary Materials

The following dataset was submitted as supplementary material: “sensors-18-00458-supplementary”. It contains all the preprocessed, gathered sensor data for each participant, including the baseline values for each signal.
